# Salivary Oral Microbiome of Children With Juvenile Idiopathic Arthritis: A Norwegian Cross-Sectional Study

**DOI:** 10.3389/fcimb.2020.602239

**Published:** 2020-11-04

**Authors:** Paula Frid, Divyashri Baraniya, Josefine Halbig, Veronika Rypdal, Nils Thomas Songstad, Annika Rosèn, Johanna Rykke Berstad, Berit Flatø, Fadhl Alakwaa, Elisabeth Grut Gil, Lena Cetrelli, Tsute Chen, Nezar Noor Al-Hebshi, Ellen Nordal, Mohammed Al-Haroni

**Affiliations:** ^1^ Department of ENT, Division of Oral and Maxillofacial Surgery, University Hospital North Norway, Tromsø, Norway; ^2^ Public Dental Service Competence Centre of North Norway, Tromsø, Norway; ^3^ Department of Clinical Medicine, UiT the Arctic University of Norway, Tromsø, Norway; ^4^ Oral Microbiome Laboratory, Kornberg School of Dentistry, Temple University, Philadelphia, PA, United States; ^5^ Department of Clinical Dentistry, UiT the Arctic University of Norway, Tromsø, Norway; ^6^ Department of Pediatrics and Adolescence Medicine, University Hospital of North Norway, Tromsø, Norway; ^7^ Department of Clinical Dentistry, University of Bergen, Bergen, Norway; ^8^ Department of Oral and Maxillofacial Surgery, Haukeland University Hospital, Bergen, Norway; ^9^ Department of ENT, Division of Oral and Maxillofacial Surgery, Oslo University Hospital, Oslo, Norway; ^10^ Department of Rheumatology and Infectious Diseases, Institute of Clinical Medicine, University of Oslo, Oslo, Norway; ^11^ Department of Rheumatology, Oslo University Hospital, Oslo, Norway; ^12^ Department of Computational Medicine and Bioinformatics, University Michigan, Ann Arbor, MI, United States; ^13^ Center of Oral Health Services and Research (TkMidt), Trondheim, Norway; ^14^ Department of Microbiology, Forsyth Institute, Cambridge, MA, United States

**Keywords:** juvenile idiopathic arthritis, salivary microbiome, next generation sequencing (NGS), oral health, 16S rRNA

## Abstract

**Background:**

The oral microbiota has been connected to the pathogenesis of rheumatoid arthritis through activation of mucosal immunity. The objective of this study was to characterize the salivary oral microbiome associated with juvenile idiopathic arthritis (JIA), and correlate it with the disease activity including gingival inflammation.

**Methods:**

Fifty-nine patients with JIA (mean age, 12.6 ± 2.7 years) and 34 healthy controls (HC; mean age 12.3 ± 3.0 years) were consecutively recruited in this Norwegian cross-sectional study. Information about demographics, disease activity, medication history, frequency of tooth brushing and a modified version of the gingival bleeding index (GBI) and the simplified oral hygiene index (OHI-S) was obtained. Microbiome profiling of saliva samples was performed by sequencing of the V1-V3 region of the 16S rRNA gene, coupled with a species-level taxonomy assignment algorithm; QIIME, LEfSe and R-package for Spearman correlation matrix were used for downstream analysis.

**Results:**

There were no significant differences between JIA and HC in alpha- and beta-diversity. However, differential abundance analysis revealed several taxa to be associated with JIA: *TM7-G1*, *Solobacterium* and *Mogibacterium* at the genus level; and *Leptotrichia* oral taxon 417, *TM7-G1* oral taxon 352 and *Capnocytophaga* oral taxon 864 among others, at the species level. *Haemophilus* species, *Leptotrichia* oral taxon 223, and *Bacillus subtilis*, were associated with healthy controls. *Gemella morbillorum*, *Leptotrichia* sp. oral taxon 498 and *Alloprevotella* oral taxon 914 correlated positively with the composite juvenile arthritis 10-joint disease activity score (JADAS10), while *Campylobacter* oral taxon 44 among others, correlated with the number of active joints. Of all microbial markers identified, only *Bacillus subtilis* and *Campylobacter* oral taxon 44 maintained false discovery rate (FDR) < 0.1.

**Conclusions:**

In this exploratory study of salivary oral microbiome we found similar alpha- and beta-diversity among children with JIA and healthy. Several taxa associated with chronic inflammation were found to be associated with JIA and disease activity, which warrants further investigation.

## Introduction

Juvenile idiopathic arthritis (JIA) is the most common chronic rheumatic disease in children, with an annual incidence of 1 to 2 per 1000 children (Moe and Rygg, 1998; Berntson et al., 2003). The pathogenesis of JIA remains unknown, although environmental triggers of disease in genetically predisposed individuals have been suggested (Cho and Blaser, 2012). Infectious agents are suspected to be environmental triggers for JIA and one of several possible mechanisms is molecular mimicry between bacterial molecules and self-antigens. It is well-known that host-microbe interaction is important for recognition and development of the immune system (Rossi et al., 2013). The human microbiome includes the collective genomes of the microbiota, which is the term for all microbes in the body (Cho and Blaser, 2012). A dysbiotic imbalance in the host microbiota might contribute as a potential trigger in the development of immune-mediated diseases, including JIA (Scher and Abramson, 2011). The gut microbiota in children with JIA is reported to differ from healthy individuals (Stoll et al., 2014; Di Paola et al., 2016; Stoll et al., 2016; Tejesvi et al., 2016; Aggarwal et al., 2017; Stoll et al., 2018; De Filippo et al., 2019; Dong et al., 2019; Van Dijkhuizen et al., 2019), where a lower abundance of Firmicutes and a higher abundance of Bacteroidetes were found in the gut microbiota of patients with oligoarticular and polyarticular rheumatoid factor (RF) negative new-onset JIA (Tejesvi et al., 2016). Higher abundance of Bacteroides is also seen in enthesitis-related-arthritis (Aggarwal et al., 2017). In all studies on the gut microbiome in JIA, however, no single species has been identified and different studies show changes in different taxa (Stoll et al., 2014; Di Paola et al., 2016; Stoll et al., 2016; Tejesvi et al., 2016; Aggarwal et al., 2017; Stoll et al., 2018; Dong et al., 2019; Van Dijkhuizen et al., 2019). These studies suggest that dysbiosis in the microbiota with overabundance in pathogenic microbes, may result in dysregulation of the immune system through disruption of the integrity of mucosal barrier and altered interaction with gut immune cells (Majumder and Aggarwal, 2020). Aberrations in mucosal homeostasis may be associated with the increased bacterial urease activity, reportedly found in fecal samples of JIA patients as compared to healthy controls. The increase of urease activity is hypothesized to be the result of alterations in the anaerobic bacterial environment (Malin et al., 1996). It is still an open question whether the observed microbial dysbiosis is a cause or an effect of the disease.

The oral microbiome has been proposed to play a role in rheumatoid arthritis by contributing to systemic inflammation (Lorenzo et al., 2019), and may also play a similar role in JIA. Furthermore, dysbiosis and periodontitis have been found to be associated to increased severity of rheumatoid arthritis (Scher et al., 2012). Gingivitis, i.e. gingival bleeding, which is a reversible inflammation of the gingiva caused by dental biofilm accumulation is a prerequisite for progression to periodontitis (Murakami et al., 2018). Gingival inflammation is in some studies found to be higher in individuals with JIA compared to healthy (Welbury et al., 2003; Ahmed et al., 2004) It has been suggested that bacteria found in the saliva are representative for the oral bacteria associated with the different oral mucosal surfaces. So far, there have been no attempts to characterize the salivary oral microbiome associated with JIA with the next generation sequencing method (NGS). *The aim of this study was therefore to investigate the oral microbiome in saliva of children with JIA and relate it to the disease activity including gingival inflammation.*

## Materials and Methods

### Study Design and Subject Recruitment

The present cross-sectional study is a project within NorJIA (Norwegian JIA Study – Imaging, oral health and quality of life in children with juvenile idiopathic arthritis (JIA)), a larger Norwegian prospective multicenter cohort study on JIA registered in Clinical Trials.gov (NCT03904459). The clinical and demographic data was collected between November 2015 and December 2018 at the Department of Pediatrics and Adolescence Medicine, University Hospital North Norway (UNN), Public Dental Service Competence Centre of North Norway (PCNN), Tromso, Haukeland University Hospital Bergen, and Oslo University Hospital, Rikshospitalet, Oslo. Informed consent was collected from all study participants and the study was approved by the Institutional Medical Research Ethics Committee (2015/318). Ninety-three children in total were recruited; Fifty-nine patients with JIA: patients with JIA and newly diagnosed temporomandibular joint (TMJ) arthritis (n = 15) at the departments above, consecutive patients with JIA without TMJ arthritis (n = 44) from the outpatient clinic at UNN, and healthy controls (HC; n = 34) at PCNN matched for age and gender to 34 of the patients with JIA (**Table 1**). HC were recruited from the larger multicenter study NorJIA, and consisted of children attending the regular Norwegian community dental care. The clinical characteristics of the two groups are presented in **Table 1**. Inclusion criteria for patients with JIA were fulfillment of the JIA classification criteria defined by the International League of Associations for Rheumatology (ILAR) (Petty et al., 2004), and age at the study visit <18 years, with or without arthritis activity in one or both TMJs. TMJ arthritis was defined as clinical symptoms and findings in addition to signs of arthritis in magnetic resonance imaging (MRI). Patients on antibiotics prior to sampling were excluded.

**Table 1:** Demographic and disease activity characteristics among children with juvenile idiopathic arthritis (JIA) and healthy controls (HC).

	JIA (n = 59)	HC (n = 34)	Cut-off	P-value*
**Demographic characteristics**				
Female, number (%)	43 (73)	27 (79)		0.48^a^
Age at sampling, years	12.6 ± 2.7	12.3± 3.0		0.65^b^
Age at onset	6.0 (2.0–10.0)			–
**Geographics**, number (%)				
Troms county	34 (58)	34 (100)		–
Finnmark county	17 (29)			–
Nordland county	5 (9)			–
Eastcoast county	2 (3)			–
Westcoast county	1 (2)			–
**Disease duration, years**	5.0 (3.0–10.0)			–
**JIA category, number (%)**				
Persistent oligoarthritis	11 (19)			–
Extended oligoarthritis	13 (22)			–
Polyarthrtitis RF positive	3 (5)			–
Polyarthrtitis RF negative	15 (25)			–
Systemic arthritis	0 (0)			–
Psoriatic arthritis	3 (5)			–
Enthesitis related arthritis	7 (12)			–
Undifferentiated arthritis	7 (12)			–
**GBI, %** (IQR)	22 (6–44) (n = 44)	6 (0–11) (n = 25)	>10	0.00^b^
**OHI-S** (IQR)	0.5 (0.3–0.8) (n = 43)	0.3 (0.0–0.4) (n = 25)		0.00^b^
**DI-S** (IQR)	0.5 (0.3–0.8) (n = 43)	0.3 (0.0–0.3) n = 25)		0.00^b^
Disease activity variables**				
JADAS10	12.8 (7.6–18.0) n = 48			
Patients with active disease, number (%)	44 (74)			–
Patients with active joints, number (%)	23 (39)			–
Patients with TMJ arthritis, number (%)	15 (25)			–
Patients with IACs to the TMJ, number (%)	8 (13)			–
Numberof active joints	0.0 (0.0–1.0)			
MDgloVAS	2.5 (1.0–5.0) (n = 58)			
PRgloVAS	2.5 (0.5–4.0) (n = 49)			
HLA-B27 positive, number (%)	20 (36.4) (n = 55)			–
Rheumatoid factor positive, number (%)	1 (2.0) (n = 51)			–
**Type of Medication**				
No DMARDs, number (%)***	15 (25)			
Methotrexate, number (%)	20 (34)			–
Biologics combination, number (%)****	24 (41)			–

Values are the median (IQR) unless indicated otherwise. ^a^Chi-square test. ^b^Wilcoxon-Mann-Whitney test. *P <0.05 for statistical significance. **Remission status according to the ACR provisional remission criteria (Wallace et al., 2011); ***NSAIDs and/or IACs; ****Current or previous use alone or in combination with other DMARDs; JIA, juvenile idiopathic arthritis; GBI, gingival bleeding index; OHI-S, simplified oral hygiene index; DI-S, simplified debris index; JADAS10, the composite juvenile arthritis10-joint disease activity score; TMJ, temporomandibular joint; MDgloVAS, medical doctor global evaluation of overall disease activity on a 10-cm visual analogue scale; IACs, intraarticular corticosteroid injections; DMARDs, disease modifying antirheumatic drugs.

### Demographics and Assessment of JIA Disease Activity

Patient demographics, subtype of JIA, duration and onset of JIA, medication, general clinical examination, measures of disease activity and severity were collected by three experienced pediatric rheumatologists calibrated through regular meetings in the study period with clinical variables thoroughly discussed and defined in a common study protocol based on the Temporomandibular joint Juvenile Arthritis Working group (TMJaw) recommendations (Stoustrup et al., 2019). Number of active joints was defined according to the general definition of arthritis: swelling within a joint or limitation in the range of joint movement with joint pain or tenderness (Filocamo et al., 2011), while TMJ arthritis was based on both clinical signs and symptoms, and MRI imaging. Patient-reported global assessment of overall well-being (PRgloVAS) and patient-reported pain (PRpainVAS) within the last week on a 10-cm visual analogue scale (VAS) were collected. On this scale, 0 indicates no activity/no pain/best global health, and 10 indicate the maximum activity/worst pain/poorest global health, respectively. A routine complete blood cell count, including rheumatoid factor (RF) and human leukocyte antigen B27 (HLA-B27) was registered. The composite juvenile arthritis disease activity score (JADAS10, range from 0 to 40) was calculated as the simple sum of the medical doctor global evaluation of overall disease activity on a 10-cm visual analogue scale (VAS), MDgloVAS (range 0–10), PRgloVAS (range 0–10), active joint count (up to maximum 10 joints), and the erythrocyte sedimentation rate (ESR) (normalized to 0–10) (Consolaro et al., 2009; Consolaro et al., 2011). Inactive disease was defined according to the ACR provisional criteria requiring all the following: 1) no active joints; 2) no fever, rash, serositis, splenomegaly or generalized lymphadenopathy attributable to JIA; 3) no active uveitis; 4) normal ESR or C-reactive protein (CRP); 5) MDgloVAS = 0; and 6) duration of morning stiffness of ≤15 minutes (Wallace et al., 2011).

### Intraoral Examination and Collection of Saliva

A modified version of the Gingival bleeding index (GBI) (Ainamo and Bay, 1975) and the Simplified Oral Hygiene Index (OHI-S) (Greene and Vermillion, 1964) were used with 6 index teeth for two reasons: 1) The youngest children had transitional dentitions with premolars and canines not yet erupted. Central incisors and first molars being the first permanent teeth to be erupted were therefore chosen as index teeth. 2) For the youngest children it was considered too exhausting and time consuming to investigate the complete dental set during the oral examination. For GBI, a dental probe was carefully applied without any pain, in the upper part of the gingival sulcus, and then removed without doing a horizontal movement along the tooth surface. Bleeding on probing within 10 seconds was registered. Angulation of the dental probe of 60 degrees to the vertical axis of the tooth was applied if possible. The mesial, distal and central site of the buccal surface of the index teeth 16, 26, 11, and 31 were chosen together with the lingual surface of the index teeth 36 and 46. The number of bleeding tooth sites were divided to the total number of tooth sites examined and finally presented as the mean percentage (%), range 0% to 100% where a higher percentage represents a worse score in bleeding. Gingival inflammation was diagnosed according to a gingival bleeding index cut-off score ≥ 10% (Trombelli et al., 2018).

OHI-S is a sum score of simplified-debris index (DI-S) and simplified-calculus index (CI-S) and is presented as a mean score index, where a higher index represents a worse score in OHI-S. DI-S and CI-S are the buccal scores + the lingual scores divided by the total number of examined surfaces. OHI-S was not calculated in children with fixed orthodontic appliances and subgingival status was not evaluated. All children ≥12 years filled out an oral health related questionnaire and the parents/proxies filled out for children <12 years. One of the questions was; how often do you brush your teeth: 1) Never 2) Most days 3) Once daily 4) Twice daily or more.

Two calibrated specialists in oral and maxillofacial surgery and pediatric dentistry (PF, JH) collected before oral examination, unstimulated whole saliva for 6 minutes and paraffin chewing stimulated whole saliva for 3 minutes, according to a standardized protocol (i.e. restrictions to food and drinks 2 h prior to sampling). Furthermore, medications taken the same day or the day before sampling was recorded. Only SWS were used for microbial analyses. After collection, each saliva sample was aliquoted and placed in a −80°C freezer until further analyses.

### DNA Extraction

Seven hundred and fifty microliters from each SWS sample was mixed with an equal amount of phosphate buffer saline (PBS) and spun down at 9600 g for 5 minutes, before the supernatant was carefully removed. The pellet was resuspended in 155 mL PBS and 25 mL MetaPolyzyme multilytic enzyme mix (Zigma-Aldrich, USA) and incubated on a 37^°^C heat block for 4 h, for digestion of the bacterial cell wall. The digests were then transferred to a QIAcube (Qiagen, Hilden, Germany) for DNA extraction using preprogramed protocol using the QIAamp DNA Mini Kit (Qiagen, Germany) with 100 μL elution volume. The quality of the isolated DNA (high molecular weight and non-fragmented DNA) was assessed by running extracted DNA samples on agarose gel (1%) with 1 kb ladder (Termo Fisher Scientific, Invirtogen, USA). The amount of yield DNA was then measured using Invitrogen Qubit 3.0 Fluorometer (Termo Fisher Scientific, Invirtogen, USA) according to the manufacturer’s instructions.

### 16S rRNA Sequencing and Bioinformatic Analysis

16S rRNA gene library preparation and sequencing were done at the Australian Center for Ecogenomics (Brisbane, Australia) as detailed previously (Al-Hebshi et al., 2017a). Briefly, the V1-3 region was amplified using the degenerate primers 27FYM (Frank et al., 2008) and 519R (Lane et al., 1985) in standard PCR conditions. The amplicons (~ 520 bp) were purified and indexed with unique 8-base barcodes in a second PCR. The tagged libraries were then pooled together in equimolar concentrations and sequenced using MiSeq v3 2 × 300 bp chemistry (Illumina, USA). Preprocessing of data (merging of reads, primer trimming, quality-filtration, alignment and chimera removal) was performed as detailed elsewhere (Al-Hebshi et al., 2017b). The high quality, merged reads were assigned species-level taxonomies using our BLASTn-based algorithm (Al-Hebshi et al., 2015; Al-Hebshi et al., 2017b). The resultant microbial profiles were used as input to QIIME (Quantitative Insights Into Microbial Ecology) software package version 1.9.1 (Caporaso et al., 2010) for downstream analysis including subsampling, generation of taxonomy plots/tables and rarefaction curves, and calculation of species richness, coverage, alpha diversity indices and beta diversity distance matrices. Principal component analysis (PCoA) was used for clustering samples based on overall microbial similarity, while Linear discriminant analysis (LDA) effect size (LEfSe) (Segata et al., 2011) was used to detect differentially abundant taxa between the groups.

### Statistical and Bioinformatic Analyses

For description of clinical and demographic data, median (IQR), mean (standard deviation) and frequencies (percentage) were used. Different disease characteristics and associations between patients with JIA and HC were analyzed by chi-square test or Fisher’s exact test for categorical variables and Student’s t-test for continuous variables if reasonably normally distributed, otherwise Man-Whitney U test was used. A multivariable logistic regression analysis was performed to adjust for OHI-S, age and gender in the association between JIA and GBI. A *P*-value ≤0.05 was considered statistically significant for clinical parameters. For testing correlation between species and measures of disease activity (JADAS10, number of active joints), p-values were adjusted for multiplicity with Benjamini-Hockberg method (FDR ≤0.1). To assess the association between the bacterial profiles and disease activity (JADAS10 and number of active joints) a Spearman correlation matrix was computed with R package. Correlations with P-value ≤ 0.01 were considered significant.

## Results

### Demographic and Disease Activity Parameters

Demographics and disease activity parameters for the group with JIA, and HC are given in **Table 1**. There was a female predominance in both groups, and RF negative polyarthritis was the most common category among children with JIA (25%). The simplified oral hygiene index (OHI-S) was significantly higher among children with JIA with median 0.5 (IQR 0.3–0.7) compared to median 0.3 (IQR 0.0–0.4) in HC. Also, the simplified debris-index (DI-S) was higher in JIA (**Table 1**). There was also significantly higher modified gingival bleeding index (GBI) in the group with JIA with median 22 (Interquartile range (IQR)) 6–40) % compared to HC with median 6.0 (IQR 0–11) % but no association was found between JIA and GBI when adjusting for OHI-S (**Supplementary Table 1**). Within the JIA group no significant differences in GBI were found between patients without DMARDs, on methotrexate or on biologics, with GBI median 10 (IQR 10–30) %, median 20 (IQR 10–40) %, and median 30 (IQR 20–40) %, respectively. There were no difference in frequency of tooth brushing between children with JIA and HC, in JIA 37 of 47 (79%) and in HC 23 of 32 (72%) brushed their teeth twice or more daily. No differences were seen in toothbrush frequency, OHI-S, DI-S, or GBI between children with and without TMJ-arthritis. Restrictions according to food and drinks 2 h prior to saliva sampling were taken in 41 of 93 patients (44%). In 10 of 93 (11%) no restrictions were taken, and in 42 of 93 (45%) information on restrictions were not available. Among the 93 participants 16 reported intake of oral medications other than disease modifying antirheumatic drugs (DMARDs) such as non-steroidal anti-inflammatory drugs (NSAIDs) and/or other medications. Three participants reported intake of oral or parenteral methotrexate, and 6 parenteral biologic agents, while the remaining 68 participants reported no intake of medication the same day or the day before saliva sampling.

### Sequencing and Data Processing Statistics

The raw data has been deposited and is publicly available from SRA (# PRJNA605805). A total of ~8.2 million sequences were obtained (range of 30,113–526,987 reads per sample), of which about 85% were successfully merged; however, only 35% were retained after quality filters and 20% after chimera removal. Of the high-quality, non-chimeric reads, 88% could be assigned species-level taxonomy (mean of 15 360± 18 368 reads per sample). Details of the reads statistics before and after each quality control step are provided in **Supplementary dataset 1**.

### Overall Microbial Profile

**Figure 1:**
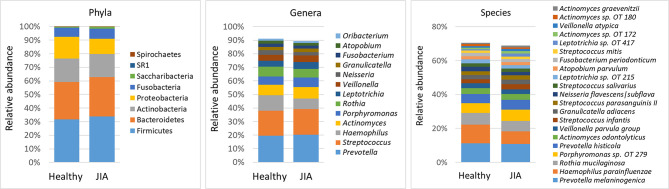
Microbiological profiles. DNA extracted from saliva was sequenced for the V1-V3 region of the 16S rRNA gene using paired-end chemistry. The generated reads were merged, quality-filtered and classified to the species level using a BLASTn-based algorithm. The stacked bars show the average relative abundances of all phyla and top genera and species (those with relative abundance ≥ 1%) identified in the study groups. OT, oral taxon.

Using a minimum count of 100 reads per species as cutoff, a total of 216 bacterial species belonging to 58 genera and 8 phyla were identified across all samples; the relative abundances and detection frequencies of these taxa in each sample is provided in **Supplementary dataset 2**, **3** and **4** respectively. On average, 134 species (range, 90–186) and 45 genera (range, 32–58) were detected per subject. Fifty-seven species and 29 genera were identified in more than 90% of the samples and can be defined as core salivary taxa. The average relative abundances of all phyla, top genera and species (those present at an average abundance of ≥ 1% in the control group) in each of the study groups are shown in **Figure 1**. Firmicutes, Bacteroidetes, Actinobacteria, Proteobacteria, and Fusobacteria were the major phyla in order accounting for more than 98% of the reads. Thirteen genera accounted for 90% of the average microbiome, with *Prevotella*, *Streptococcus*, *Haemophilus, Actinomyces*, *Porphyromonas* and *Rothia* alone making up ~ 70%. At the species level, *Prevotella melaninogenica, Haemophilus parainfluenzae, Rothia mucilaginosa, Porphyromonas* sp. *oral taxon 279, Prevotella histicola, Actinomyces odontolyticus* were the most abundant species, constituting around 40% of the microbiome on average.

### Bacterial Diversity and Differentially Abundant Species

**Figure 2:**
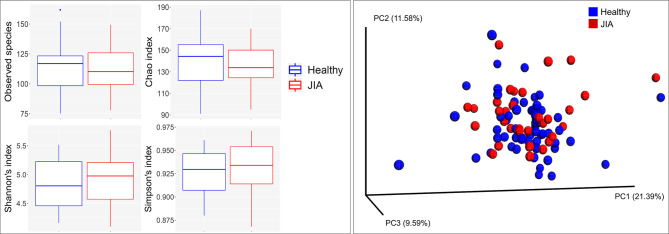
Species richness and diversity. Taxonomic profiles were rarified and used to calculate observed richness, expected richness (alpha diversity index; Chao index), evenness measure (alpha diversity index; Shannon’s and Simpson’s) and distance matrices employing standard QIIME scripts. Left: Box and whisker plots of species richness and aloha diversity in each group. Differences were not significant by Mann–Whitney U test. Right: Clustering of samples with PCoA based on abundance Jaccard distance matrix. Plots were generated with QIIME and R Package.

**Figure 3:**
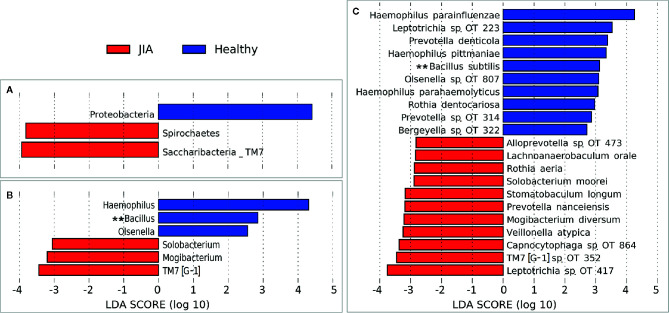
Differentially abundant taxa. **(A)** Phyla, **(B)** Genera and **(C)** species that showed significant differences in relative abundance between the two study groups as identified by linear discriminant analysis (LDA) effect size analysis (LEfSe) – 2.5 LDA score cutoff. OT, oral taxon. **FDR ≤ 0.1 (Benjamini-Hochberg method).

There were no significant differences between children with JIA and the healthy group in the number of species (i.e. alpha diversity, species richness), or in PCoA (i.e. beta diversity, the ratio between the two groups) as shown in **Figure 2**. Differential abundance analysis revealed significant differences (**Figure 3**). At the phylum level, JIA was associated with enrichment of Spirochaetes and Saccharibacteria and depletion of Proteobacteria. Genera *TM7-G1*, *Solobacterium* and *Mogibacterium* were associated with JIA, while *Haemophilus* and *Bacillus* were associated with healthy subjects (**Figure 3B**). *Haemophilus parainfluenzae*, *Leptotrichia* *species oral taxon 223, Haemophilus pittmanae*, *Prevotella denticola* and *Bacillus subtilis* were key bacterial species associated with the healthy group, whereas the JIA group showed higher abundance of 11 bacterial species of which *Leptotrichia species oral taxon 417, TM7 G1*, *Capnocytophaga* *species oral taxon 864*, *Veilonella atypica* and *Mogibacterium diversum* were most enriched (**Figure 3C**).

**Figure 4:**
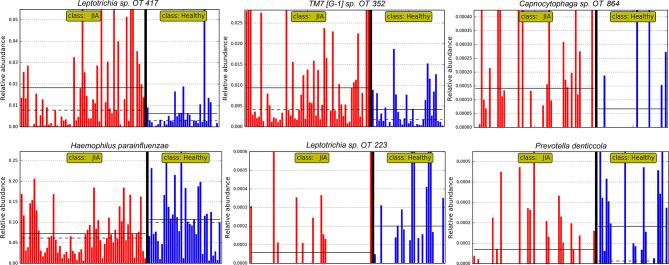
Per sample abundance plots. Relative abundances of top six differentially abundant species (based on LDA score) in individual samples. OT, oral taxon. FDR ≤ 0.1 (Benjamini-Hochberg method).

The relative abundances of the top six differentially abundant taxa (based on LDA score) are shown for individual samples in **Figure 4**. These microbial associations between the JIA and the healthy group were independent of the differences between the two groups in GBI. The microbial associations with the GBI are shown in **Supplementary Figure 1**. Notably, after adjustment of p-values for multiple comparisons with Benjamini-Hochberg method only genus *Bacillus* and *B. subtilis* maintained a false discovery rate (FDR) of ≤ 0.1 and the per sample relative abundance plot for *B. subtilis* is shown in **Supplementary Figure 2**.

### Microbial Association With Disease Activity

**Figure 5:**
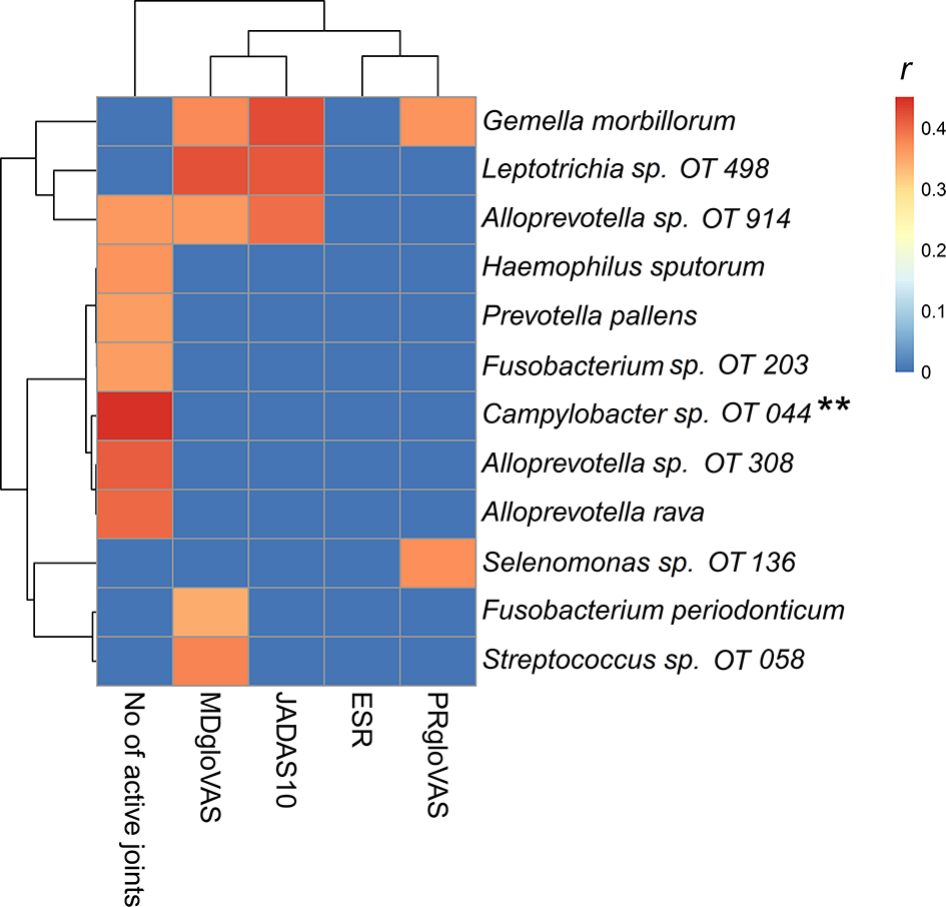
Heatmap of the microbial association with disease activity. A Spearman correlation matrix was computed using R package. Correlations with P-value ≤ 0.01 were considered significant. The r-value for nonsignificant correlations was set to zero (blue on the heatmap). OT, oral taxon. **FDR ≤ 0.1 (Benjamini-Hochberg method). PRgloVAS; patient reported global assessment of well-being; ESR, erythrocyte sedimentation rate; JADAS10, the composite juvenile idiopathic arthritis 10-joint disease activity score; MDgloVAS, the medical doctor global evaluation of disease activity.

Correlation analysis revealed significant association between a group of bacteria species and disease activity (**Figure 5**). *Gemella morbillorum*, *Leptotrichia* sp. oral taxon 498 and *Alloprevotella* oral taxon 914 correlated positively with the composite juvenile arthritis 10-joint disease activity score **(**JADAS10), primarily through their association with the medical doctor global evaluation of disease activity (MDgloVAS). *G. morbillorum* also correlated with patient reported global assessment of well-being (PRgloVAS). Several species correlated positively with the number of active joints but *Campylobacter* oral taxon 44 showed the strongest association and was the only species that maintained FDR ≤ 0.1 when *P*-values were adjusted for multiplicity with the Benjamini-Hockberg method.

**Figure 6:**
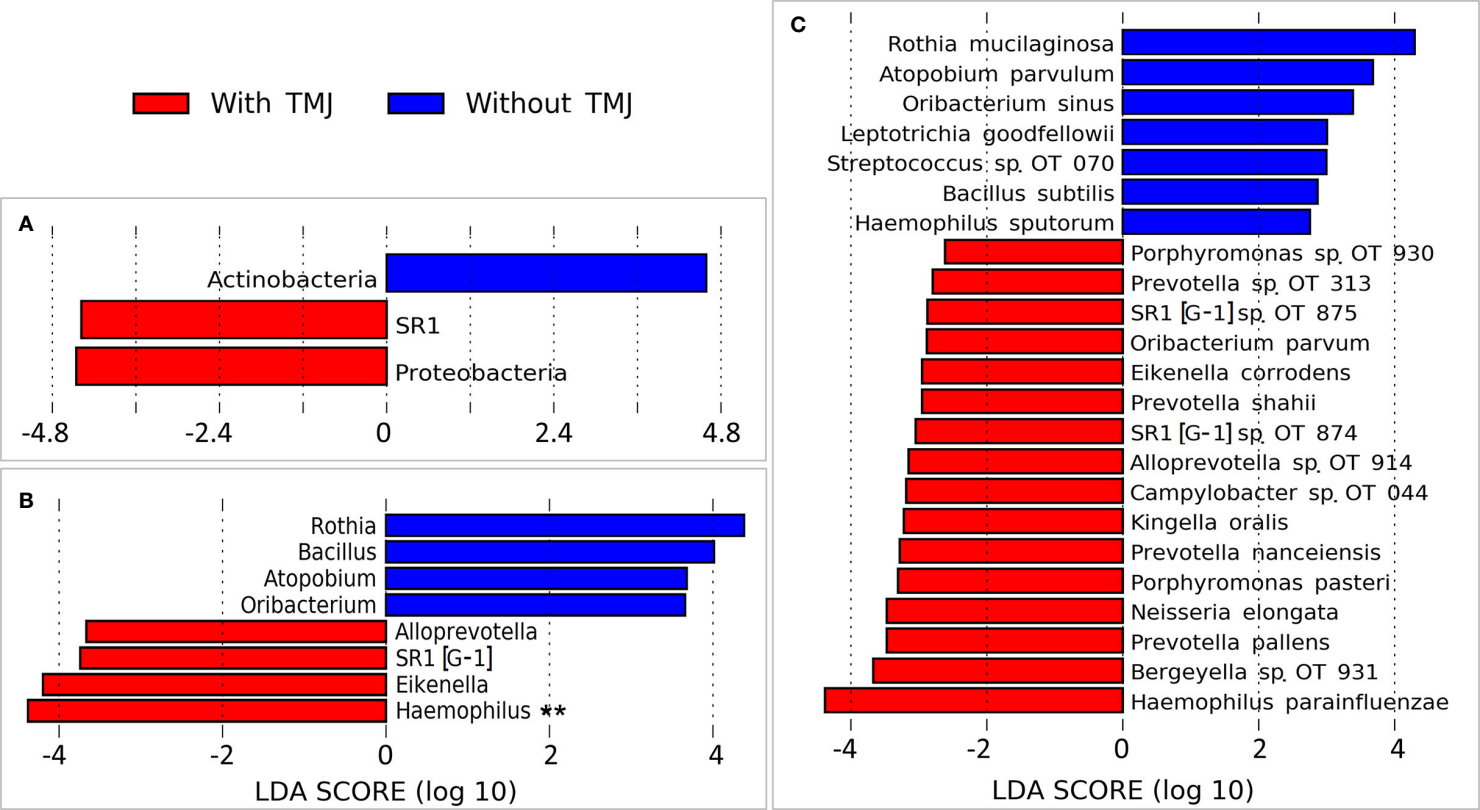
Differentially abundant taxa by TMJ arthritis. **(A)** Phyla, **(B)** Genera and **(C)** species that showed significant differences in relative abundance between the JIA subjects with and without TMJ involvement, as identified by linear discriminant analysis (LDA) effect size analysis (LEfSe). 2.5 LDA score cutoff. OT, oral taxon. **FDR ≤ 0.1 (Benjamini-Hochberg method).

Children with JIA and TMJ arthritis showed different microbial associations compared to JIA subjects without TMJ arthritis (**Figure 6**). *Haemophilus parainfluenzae, Prevotella pallens, actinomyces species oral taxon 180* were the top species differentially enriched in JIA with TMJ subjects. *Campylobacter* oral taxon 44 also showed an association with TMJ arthritis (**Supplementary Figure 3**), although opposed to its association with the number of active joints, it did not maintain FDR ≤ 0.1. *Rothia mucilaginosa, Atopobium parvulum* and *Oribacterium sinus* were significantly more abundant in children with JIA without TMJ arthritis.

## Discussion

This study is to our knowledge, the first to examine the salivary oral microbiome in JIA patients with the NGS method. Different methods are suggested for collecting saliva samples which might alter the NGS sequencing results but it seems that the overall microbial composition of saliva is not significantly affected (Lim et al., 2017; Mascitti et al., 2019). Salivary microbiome is a good representative of bacteria found in many oral mucosal sites such as cheeks, tongue, gingiva, especially when studying inflammatory diseases rather than subgingival plaque, which is rather a site-specific medium for the bacteria associated with the periodontium.

The oral environment is a complex environment that contains distinct microbial niches each with its distinct microbial inhabitant in mucosal membranes and subgingival plaque. Sampling of saliva has been used when studying dysbiosis of salivary microbiota in inflammatory bowel disease  (Said et al., 2014), and sampling by tongue and buccal mucosal brushings have been used in ulcerative colitis  (Docktor et al., 2012).

In our study, saliva samples were directly aliquoted and immediately frozen before further processing. Other collection methods for sampling of oral microbiota are available and appear to be stable concerning bacterial diversity at ambient temperature after 4 to 7 days, such as mouthwash sampling methods (Vogtmann et al., 2019). Some distinct differences in relative abundance of specific microbial taxa are seen between these methods compared to samples that are frozen immediately (Vogtmann et al., 2019), indicating that direct freezing of samples may still be the best choice.

A majority of the children with JIA were females diagnosed with either oligoarthritis persistent or RF negative polyarthritis in accordance with most population-based studies, pointing to representability of our study case (Nordal et al., 2011). Another strength of this study was that a majority of the participants had no intake of systemic medication the same day or the day before saliva sampling.

A limitation of the study is that information is available only in 41 of 93 children regarding food and drinks restrictions prior to sampling. Additionally, our findings must be evaluated in the context of a limited sample size and being a cross-sectional exploratory study.

### Microbial Profile, Bacterial Diversity and Differentially Abundant Species

In our study between 90 and 186 different species were found in the salivary microbiome of the study subjects with fifty-seven species shared in more than 90% of the subjects. The core salivary microbiome in the study subjects comprised 22 species detected in all individuals. In other studies the core microbiome was found to be similar or even in a lower number (Hall et al., 2017; Hansen et al., 2018). We found no significant differences between children with JIA and HC in species richness or in PCoA, i.e. alpha or beta diversity. Differential abundance analysis, however, revealed that JIA was significantly associated with taxa associated with chronic inflammation, and that several of these species including *Campylobacter* oral taxon 44 correlated with disease activity in terms of increased number of active joints. The predominant bacterial genera found in the salivary microbiome in both JIA and healthy saliva in our study consisted of *Prevotella, Streptococcus, Haemophilus, Actinomyces*, *Porphyromonas* and *Rothia*, which is similar to other studies in both children with JIA and healthy (De Filippo et al., 2019). The microbial diversity and richness of the salivary oral microbiome between the JIA and healthy controls were comparable. This is in line with other studies that investigated the oral microbial diversity between patients with chronic inflammatory diseases, i.e. rheumatoid arthritis, and healthy controls (Scher et al., 2012; Tong et al., 2019). Differential abundance analysis revealed several taxa to be associated with or depleted in JIA. At phylum level, we found an overabundance of Spirochaetes and Saccharibacteria and depletion of Proteobacteria. This is in line with Xu et al. showing Proteobacteria (*Neisseria*) and Firmicutes (*Selenomonas*) as a healthy core salivary microbiome, together with the phyla Bacteroidetes (*Porphyromonas*). They also showed that the salivary microbial composition shifts with aging in children, and a strength of our study is age-matching between children with JIA and healthy controls (Xu et al., 2018).

At the genus level, *Haemophilus*, *Bacillius* and *Olsenella* were depleted from JIA patients while there were significant overabundance of bacteria known to be associated with chronic inflammation, such as *TM7-G1*, *Solobacterium* and *Mogibacterium* (Moore et al., 1985; Casarin et al., 2012; Hiranmayi et al., 2017). The higher abundance of *Haemophilus parainfluenzae*, *Leptotrichia* *species oral taxon 223, Haemophilus pittmanae*, *Prevotella denticola* and *Bacillus subtilis* in the healthy group in our study could highlight the importance for health of sustaining a high proportion of these species in the oral microbiome. *Bacillus subtilis* species has been reported to have a role in limiting inflammatory response by down-regulation of the pro-inflammatory interleukin-8 production and up regulation of inducible nitric oxide synthase (iNOS) protein levels (Rhayat et al., 2019). The depletion of *Haemophilus* species in salivary microbiome has also been reported in patients with rheumatoid arthritis (Zhang et al., 2015). On the other hand *Leptotrichia species oral taxon 417, TM7 G1*, *Capnocytophaga* *species oral taxon 864*, *Veilonella atypica*, and *Mogibacterium diversum* were found to be highly abundant in children with JIA. The oral taxon TM7 has been associated with chronic inflammation (Demmer et al., 2017). In line with our study, Grevich et al. found depletion of genera Prevotella (phylum Bacteroidetes) in JIA, but they report an overabundance of the genera Haemophilus and Kingella (phylum proteobacteria) in JIA, which was not found in our study (Grevich et al., 2019).

In children with JIA the saliva in those with TMJ arthritis was enriched with certain bacteria compared to those without TMJ arthritis. This finding was not explained by differences in toothbrush frequency or differences in oral hygiene indices (i.e. DI-S, OHI-S, GBI), which was similar in both groups.

The role of oral and gut microbiota in many inflammatory diseases has been suggested, and there is evidence for the role of molecular mimicry in such diseases. It has for example, been reported that a molecular mimicry between a peptide from the von Willebrand factor type A from the oral microbe *Capnocytophaga ochracea* can be attributed to the activation of the Sjögrens syndrome antigen A/Ro60-Reactive T cells (Szymula et al., 2014).

### Microbial Association to Disease Activity

Interestingly, in our study *Campylobacter* oral taxon 44 (proteobacteria at the phyla level) showed a moderate correlation with the number of joints affected in patients with JIA. *Campylobacter* has a well-known association with reactive arthritis and other inflammatory diseases in both children and adults (Lackner et al., 2019). Dong et al. found a negative correlation between disease activity and *Proteobacteria*, *Ruminococcaceae, Faecalibacterium*, or *Enterobacteriaceae* (Dong et al., 2019), in the gut microbiota in 32 patients with JIA. In line with our study, the same authors found a positive correlation between disease activity and increased abundance of phyla *Firmicutes (our study: G.morbillorum), Bacteroidetes (our study: Alloprevotella, prevotella pallens)*, and *Bacteroidaceae* at the phyla level (Dong et al., 2019).

The species found enriched in our study on the salivary oral microbiome in JIA are not the same as found in studies on the gut microbiome in JIA (Stoll et al., 2014; Di Paola et al., 2016; Stoll et al., 2016; Tejesvi et al., 2016; Aggarwal et al., 2017; Stoll et al., 2018; Dong et al., 2019; Van Dijkhuizen et al., 2019). However, in all studies disruption of microbial ecology was found with overabundance of taxa associated to chronic inflammation in JIA, and this dysbiosis may have community effects on the host more powerful than the actions of just one single microbe (Chriswell and Kuhn, 2020). Dijkhuizen et al. report dysbiosis in gut microbiota in children newly diagnosed with JIA compared to healthy controls. They also found that age and geographic origin were connected to microbiota profiles (Van Dijkhuizen et al., 2019). In rheumatoid arthritis both oral and gut dysbiosis are well described and no consistent single bacterial species appears to be the causing agent (Bergot et al., 2020). Interestingly, periodonto-pathogenic bacteria such as *Porphyromonas gingivalis* have been suggested to contribute to generation of anti-citrullinated protein antibodies (ACPAs) in rheumatoid arthritis (Moen et al., 2006; Wegner et al., 2010a; Wegner et al., 2010b). Dysbiosis and periodontitis were also found to be associated to increased severity of rheumatoid arthritis (Scher et al., 2012). Other factors reported to be associated with dysbiosis are diet, lifestyle and drug use, especially the use of antibiotics (Turnbaugh et al., 2009; Arvonen et al., 2015). Early life antibiotic use is shown to increase the risk of developing JIA later in life, and may predispose due to a shift in microbiota composition (Arvonen et al., 2015; Horton et al., 2015). In our study none of the participants were on any antibiotics on the day of sampling, but previous history of antibiotic use were not recorded.

### Gingival Inflammation and the Oral Microbiome

The significantly higher gingival inflammation found in patients with JIA compared to healthy controls is in line with many studies investigating JIA and oral health (Welbury et al., 2003; Ahmed et al., 2004; Leksell et al., 2008; Santos et al., 2015; Grevich et al., 2019). Other studies find no significant difference between JIA and healthy controls (Miranda et al., 2003; Savioli et al., 2004; Reichert et al., 2006; Feres De Melo et al., 2014; Pugliese et al., 2016; Kobus et al., 2017; Maspero et al., 2017), probably depending on different study design and measurement indices of gingival inflammation (Skeie et al., 2019). Despite higher GBI, no difference in frequency of tooth brushing was found between JIA and healthy controls in our study. However, we do not know how effective the tooth brushing was performed, some of the children with JIA have restricted wrist, finger or jaw movements that may reduce the quality of the tooth brushing. After adjusting for dental plaque and calculus (OHI-S), JIA was not found to be a predictor for gingival inflammation in terms of higher GBI. In line with other studies (Reichert et al., 2006) we found dental plaque (OHI-S) to be associated to gingival inflammation. There were no overlap between overabundant bacteria associated with GBI and those associated with JIA. Overabundance of bacteria associated with chronic inflammation in JIA could be explained by a disruption of microbial hemostasis in JIA and not by gingival inflammation in JIA.

There are some indications that the biologic agent etanercept might reduce periodontal inflammation in children with JIA (Maspero et al., 2017). In our study children with JIA had more gingival bleeding compared to healthy controls, despite immune-modulating medication. Altogether 40% were on biologic treatment either alone or in combination with methotrexate, but we found no significant differences in GBI between the different medication groups.

## Conclusion

Several taxa, including genera Solobacterium, Mogibacterium, and TM7-G1 known to be associated with chronic inflammation, were found enriched in the saliva of children with JIA and were associated with disease activity in our study. No significant difference was found in alpha- and beta-diversity compared to healthy. Prospective cohort-studies with treatment-naïve patients with new onset JIA are warranted to further elucidate the role of the oral microbiome in disease etiology and severity.

## Data Availability Statement

The raw data has been deposited and is publicly available from SRA (# PRJNA605805).

## Ethics Statement

The studies involving human participants were reviewed and approved by Institutional Medical Research Ethics Committee. Written informed consent to participate in this study was provided by the participants’ legal guardian/next of kin.

## Author Contributions

Planning of the study, analysis of the data and interpretation of the results as well as writing of the manuscript: PF, MA-H, EN, NA-H. Collection clinical data: PF, EN, JB, BF, AR, VR, NS, JH, EG, and LC. MA-H supervised the laboratory work and DB, FA TC, and NA-H the DNA sequencing analysis. Critical review and editing of manuscript: PF, EN, JB, BF, AR, VR, NS, JH, EG, LC, NA-H, and MA-H. All authors contributed to the article and approved the submitted version.

## Funding

Grethe Harbitz funds, Research funding from Troms County, Norsk Revmatikerforbund, Helse Nord Research funding and Tromsø Research Foundation and Department of Clinical Dentistry, UiT The Arctic University of Norway.

## Conflict of Interest

The authors declare that the research was conducted in the absence of any commercial or financial relationships that could be construed as a potential conflict of interest.

## Supplementary Material

Supplementary Figure 1 Heatmap of the microbial association with GBI. A Spearman correlation matrix was computed using R package. Correlations with P-value ≤ 0.01 were considered significant.

Supplementary Figure 2 Per sample abundance plots for *Bacillus subtilis*. Relative abundances of *Bacillus subtilis* in individual samples by disease status (left) and TMJ involvement status (right).

Supplementary Figure 3 Per sample abundance plots for *Campylobacter* sp. oral taxon 44. Relative abundances of *Campylobacter* sp. oral taxon 44 in individual samples by TMJ involvement status.

Supplementary Data Sheet 1 Sequencing and data processing statistics.

Supplementary Data Sheet 2 Relative abundances and detection frequencies of phyla identified in the individual samples.

Supplementary Data Sheet 3 Relative abundances and detection frequencies of genera identified in the individual samples.

Supplementary Data Sheet 4 Relative abundances and detection frequencies of species identified in the individual samples.
