# Supplementary figures and images for: Salivary Oral Microbiome of Children With Juvenile Idiopathic Arthritis: A Norwegian Cross-Sectional Study
Source: Front Cell Infect Microbiol. 2020 Nov 4;10:602239. doi: 10.3389/fcimb.2020.602239 (PMC7672027; doi:10.3389/fcimb.2020.602239)

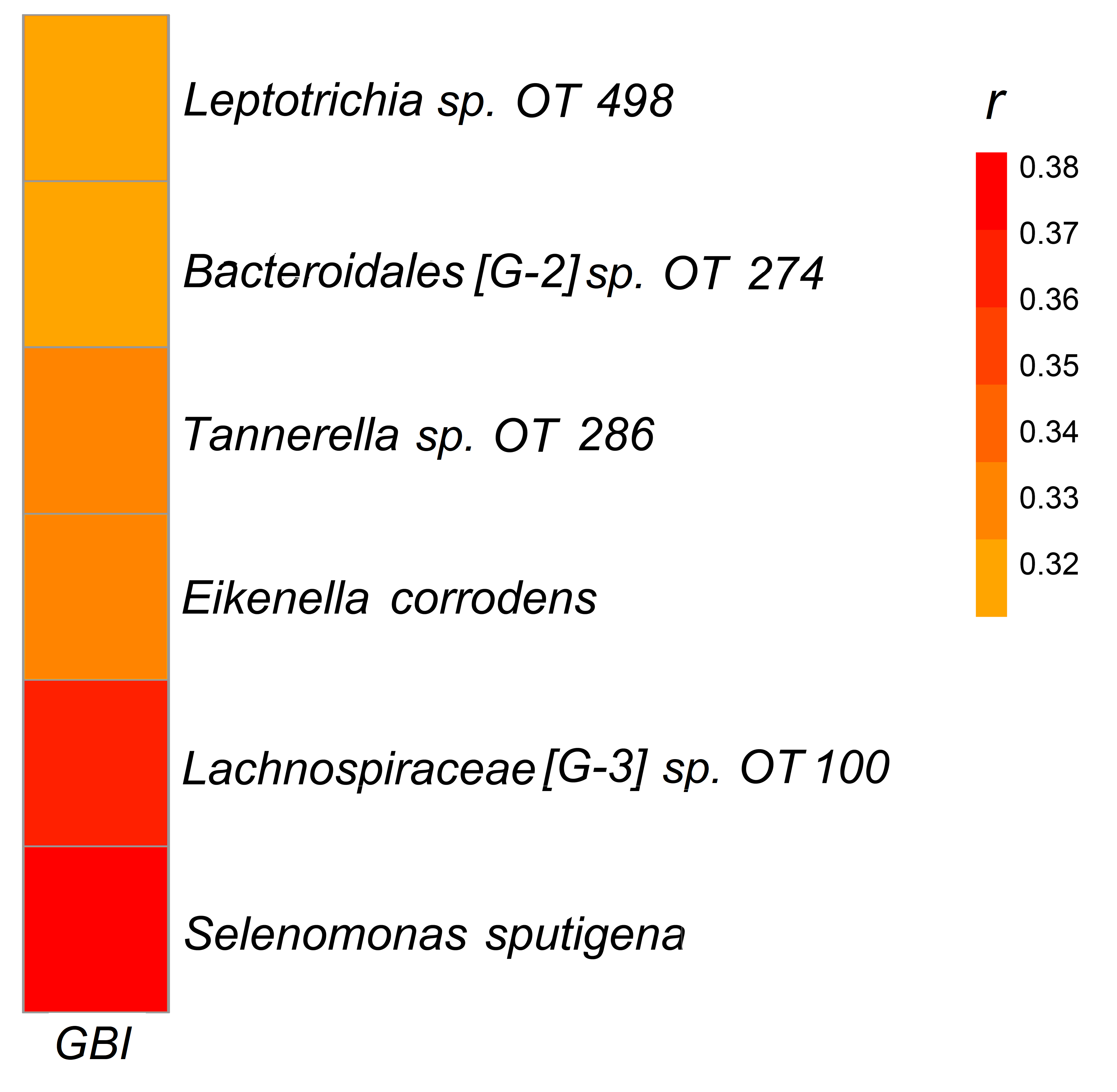

Supplement: Supplementary Figure 1 — Heatmap of the microbial association with GBI. A Spearman correlation matrix was computed using R package. Correlations with P-value ≤ 0.01 were considered significant. [file Image_1.tif]

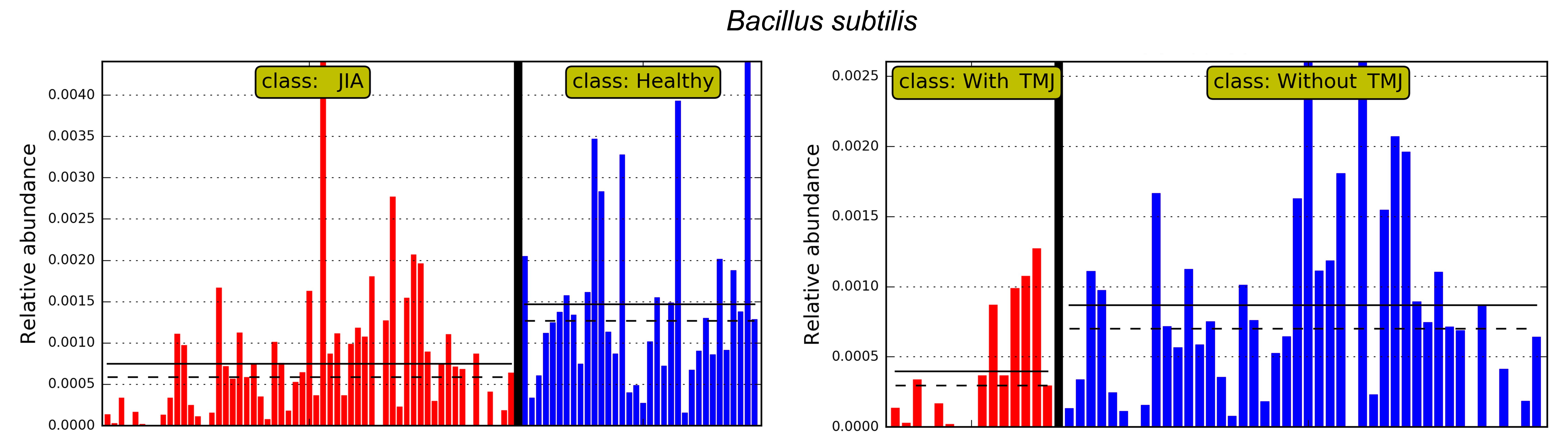

Supplement: Supplementary Figure 2 — Per sample abundance plots for Bacillus subtilis. Relative abundances of Bacillus subtilis in individual samples by disease status (left) and TMJ involvement status (right). [file Image_2.tif]

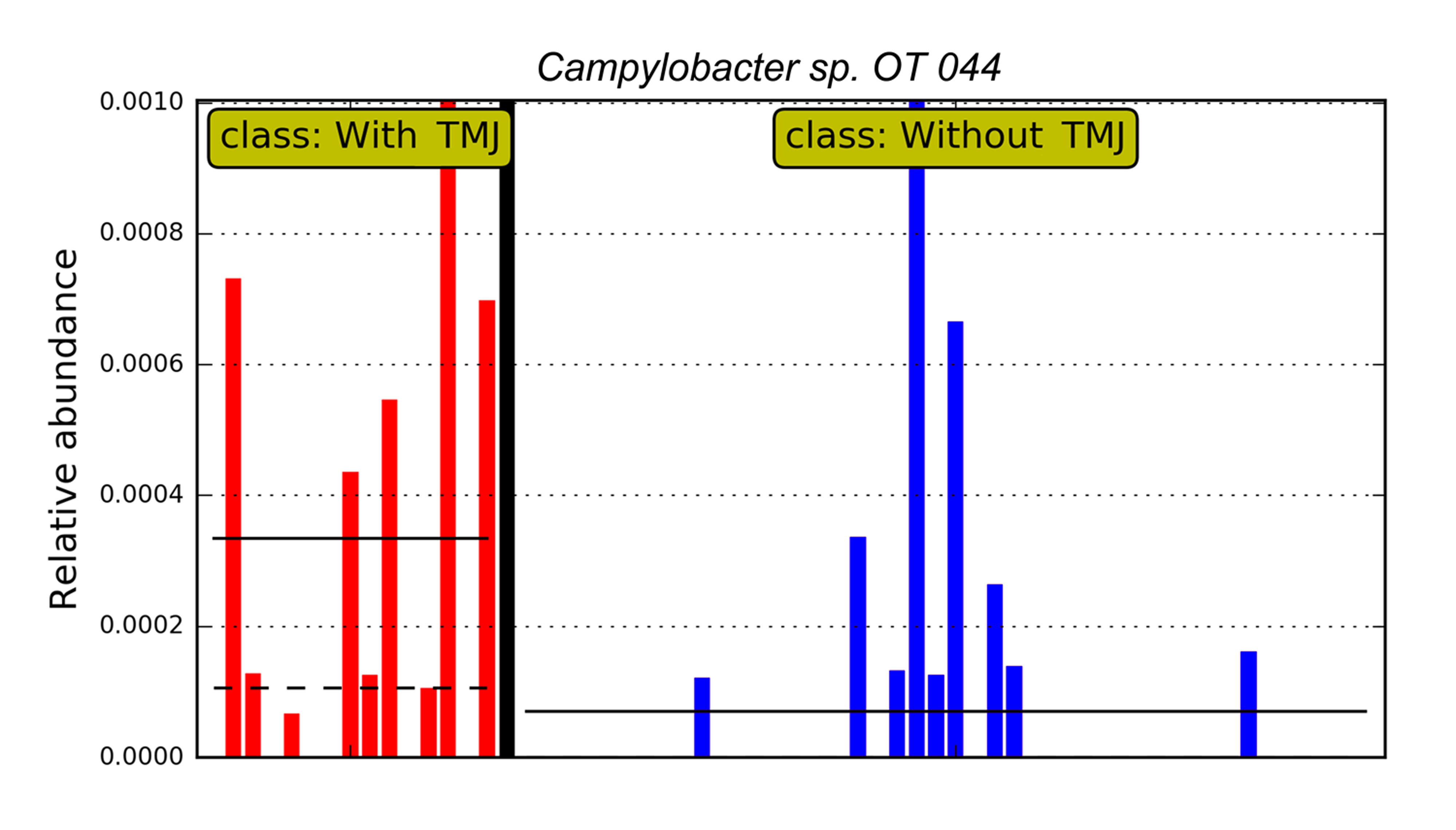

Supplement: Supplementary Figure 3 — Per sample abundance plots for Campylobacter sp. oral taxon 44. Relative abundances of Campylobacter sp. oral taxon 44 in individual samples by TMJ involvement status. [file Image_3.tif]
