# Supplementary material for: Salivary Oral Microbiome of Children With Juvenile Idiopathic Arthritis: A Norwegian Cross-Sectional Study
Source: Front Cell Infect Microbiol. 2020 Nov 4;10:602239. doi: 10.3389/fcimb.2020.602239 (PMC7672027; doi:10.3389/fcimb.2020.602239)
Supplement: Supplementary file 5 [file Table_1.docx]

**Supplementary Table 1.** Gingival inflammation (gingival bleeding index (GBI) ≥10%)* according to age, gender and JIA

|  | **Gingival inflammation (n=41)** | | | |
| --- | --- | --- | --- | --- |
|  | **Crude OR (95% CI)** | p-value | **Adjusted OR (95% CI)** | p-value |
| **JIA** | 4.8 (1.7-14.0) | 0.004 | 2.9 (0.9-9.5) | 0.07 |
| **Age** | 0.9 (0.7-1.2) | 0.651 | 0.9 (0.7-1.2) | 0.578 |
| **Gender (female)** | 0.9 (0.3-3.1) | 0.917 | 0.8 (0.2-2.8) | 0.688 |
| *Multivariable logistic regression analysis adjusted for the simplified oral hygiene index score (OHI-S). OR, odd ratio; CI, confidence interval. | | | | |
